# The CuRe Trial: Groundbreaking Innovation Raising Scientific and Ethical Questions

**DOI:** 10.15190/d.2026.7

**Published:** 2026-04-20

**Authors:** Zachary B. Sluzala

**Affiliations:** ^1^Charlotte Lozier Institute, Arlington, VA 22206, USA

**Keywords:** Spina bifida, myelomeningocele, prenatal surgery, prenatal diagnosis, fetal surgery, stem cells, biomaterials, neural tube defects.

## Abstract

Preliminary data for the Cellular Therapy for In Utero Repair of Myelomeningocele (CuRe) Trial have recently been published. These data showcase innovation in fetal surgery but introduce ethical questions regarding tissue sourcing. The CuRe Trial utilizes early gestational placental mesenchymal stromal/stem cells (PMSCs) in the repair technique. The ethical implications of this approach vary depending on whether they are sourced from miscarriage or elective abortion. The specific source of the PMSCs is unclear, but evidence suggesting that they might be sourced from elective abortion is presented, along with an overview of ethical implications if this is the case. Key questions considered include 1) whether research such as this is impacted by the recent decision by the National Institutes of Health (NIH) to cease funding of research using fetal tissue from elective abortion; 2) what ethical alternatives currently or may plausibly exist; and 3) whether scientific justifications exist for the use of tissue sourced from elective abortion over more ethical alternatives.

## SUMMARY

*1. Introduction*

*2. Ethical Questions Surrounding Stem Cell and Fetal Tissue Research*

- *2.1. PMSC Sourcing*

*3. Where Does This Leave Us?*

- *3.1. NIH Funding of HFT Research*

- *3.2. Ethical Alternatives*

- *3.3. Abortion-Sourced Tissue vs. Ethically Sourced Tissue: Why the Former?*

*4. Conclusions*

## 1. Introduction 

Mere days before the publication of our recent review (Sluzala & Furth, 2026^1^), feasibility and safety findings were published for the groundbreaking CuRe Trial^2^, demonstrating the rapidity with which the field of fetal surgery is advancing. As we discussed in the review, the CuRe Trial is an ongoing clinical trial seeking to utilize live placenta-derived mesenchymal stem cells (PMSCs) to treat spina bifida in-utero^3^. The technique involves seeding PMSCs onto an extracellular matrix, which is then applied to the myelomeningocele defect during prenatal repair. The CuRe Trial team has previously shown promising results and highlighted several success stories^4-8^. In this new report, the authors present data on six babies who underwent prenatal surgical repair. All six babies not only survived but exhibited remarkable outcomes. 

All repair sites were intact and completely healed, hindbrain herniation was reversed in each of the infants, no shunts needed to be placed for hydrocephalus, no tumor growth was observed, and only one child experienced transient respiratory distress syndrome^2^. Overall, this approach was demonstrated to be feasible and safe, and the project is slated to proceed to a phase 1/2a trial. The CuRe Trial represents the culmination of years of investigation into the use of stem cell therapies for treating spinal cord and neural tube defects and injuries^9-12^, and showcases how the open hysterotomy repair technique has changed since the initial MOMS Trial^13^. In discussing these advancements, however, there should be – as one reviewer of our narrative review aptly pointed out – “equal emphasis on unresolved challenges.” And indeed, the CuRe Trial provides an important case study on some of these challenges. Beyond those addressed in the review – translatability, limited data and sample sizes, and regulatory hurdles – one area of distinct importance to emphasize is the ethical implications of the techniques employed. In his confirmation hearing, in response to a question about fetal tissue research, Dr. Jay Bhattacharya, the current director of the NIH, clearly articulated the necessity for ethically sound clinical advancements: 

*“In public health, we need to make sure the products of the science are ethically acceptable to everybody and so having alternatives that are not ethically conflicted to fetal stem cell lines is not just an ethical issue, but it's a public health issue. We need to make sure that everyone is willing to take the kinds of progress that we make and so I'm absolutely committed to that.”* ^14^

In line with this reasoning, the National Institutes of Health (NIH) has recently announced a request for information “seeking public comment on the robustness of emerging biotechnologies to reduce or potentially replace remaining research reliance on human embryonic stem cells, which are derived from human embryos.”^15^This follows an announcement in January that NIH funds will not be permitted for research using human fetal tissue (HFT) from elective abortions^16^. The CuRe Trial findings, while undeniably significant and reflective of a recent advancement in the field of maternal-fetal medicine, also raise scientific and ethical questions regarding the sourcing and use of biomaterials such as these in clinical research. Namely, is “everyone willing to take the kinds of progress” that this trial has made? 

## 2. Ethical Questions Surrounding Stem Cell and Fetal Tissue Research 

Research employing embryonic stem cells or HFT, particularly from elective abortion, has been a longstanding subject of debate, and there have been ample (and highly successful) efforts to design and develop ethical alternatives^17-24^. Ethical concerns surrounding the use of embryonic stem cells have been perhaps most notably highlighted in two quotes by highly relevant and impactful researchers in the field. Dr. James Thomson was among the first to isolate human embryonic stem cells, and has been quoted describing the ethics of embryonic stem cell research: 

*“If human embryonic stem cell research does not make you at least a little bit uncomfortable, you have not thought about it enough. I thought long and hard about whether I would do it.”* ^25^

Dr. Shinya Yamanaka, the Nobel Prize winning pioneer of induced pluripotent stem cells (iPSCs) – a rapidly advancing alternative to human embryonic stem cell research – has also discussed his motivations for developing iPSCs in an interview, recalling looking through a microscope at a human embryo: 

*“When I saw the embryo, I suddenly realized there was such a small difference between it and my daughters ... I thought, we can't keep destroying embryos for our research. There must be another way.”* ^26^

The cells used in the CuRe Trial are *not* embryonic stem cells but are instead derived from placental tissue^27,28^. On its face, this positions the approach as less ethically problematic than use of embryonic stem cells. However, as is almost always the case in emerging biotechnologies, it is more complicated. While these cells are not embryonic stem cells, they are still derived from the placenta. The placenta, in turn, derives from the zygote and shares the same genetics as the fetus^29^. Despite this, there are those who define the placenta as either a maternal or shared organ. This question of classification is largely beyond the scope of this short editorial and has been reviewed in more detail elsewhere^30^. But it should be noted that there are several clinical and bioethical arguments for the classification of the placenta as HFT^30-35^, and the NIH does include “any human extra-embryonic cells and tissue, such as umbilical cord tissue, cord blood, placenta, amniotic fluid, and chorionic villi, if obtained from the process of elective abortion” in their definition of tissues excluded from NIH funding^36^. However, even if the placenta constitutes fetal tissue, it certainly presents an exceptional circumstance where it is possible to utilize fetal tissue without actually harming the fetus, for example, if the placenta is collected after birth or following miscarriage. With that in mind, of primary importance in understanding the ethical implications of the CuRe Trial is not whether the placenta-derived PMSCs are in fact fetal tissue, but rather, how those PMSCs are sourced. 

### 2.1. PMSC Sourcing 

The patents for these PMSCs state that “[p]lacental tissues collected include term placenta from routine births, and pre-term placentas of gestational age 12-20 weeks from terminated pregnancies.”^37-39^Collection of term placentas after birth would hypothetically position this approach as less unethical; however, it is evident that the PMSCs used in this trial were sourced from the pre-term placentas referenced in the patent, as they are referred to as “early gestational” in this and previous publications. What is not immediately evident is the specific source of the placentas. “Terminated pregnancies” could theoretically include miscarriages and/or induced abortions, and the language is not clear enough here to identify the specific source. The ethical implications of these two sources of tissue are different, and it warrants further investigation to determine which was employed for this trial. To do so, it is helpful to look back at the methods sections of previous studies pertaining to the development of the PMSC-based approach. This is where details such as this ought to appear, not only due to ethical concerns such as these, but also simply for the purposes of scientific transparency and replicability. As Farmer et al. include a timeline of relevant studies in the lead up to the CuRe Trial^2^, it is prudent to begin with these (**Table 1**).

**Table 1:** PMSC Sourcing in Articles cited in Figure 1 of Farmer et al., 2026^2^ * References included in the methods text refer to the references of *the quoted study*, not the references of this editorial.

Study	PMSC Source as per Methods Section*
Saadai et al. (2011)^40^	n/a
Saadai et al. (2013)^41^	n/a
Brown et al. (2015)^42^	n/a
Brown et al. (2015)^43^	n/a
Wang et al. (2015)^44^	“Donated early gestation placental tissue (11–17 weeks) was collected at the University of California, Davis, Medical Center.”
Brown et al. (2016)^45^	“We isolated PMSCs using an explant culture method from one randomly selected sample of early-gestation (17 weeks) placenta and one term-gestation (approx. 40 weeks) placenta.”
Lankford et al. (2017)^27^	“Discarded early gestation placental tissue (15–19 weeks) was collected at the University of California, Davis Medical Center.”
Kabagambe et al. (2018)^46^	“PMSCs were isolated from chorionic villi of a second trimester placenta and fully characterized as previously described [15]. They were derived as previously reported by Wang et al. [7].”
Vanover et al. (2019)^47^	“PMSCs were isolated from chorionic villi of one second trimester human placenta and fully characterized as previously described [17].”
Kumar et al. (2019)^28^	“We used PMSC cell banks from 3 donors as described in Lankford et al. (19), plus a cell bank from a fourth donor”
Yamashiro et al. (2020)^48^	“PMSCs were explanted from donated placentas as described by Lankford et al [9].”
Galganski et al. (2020)^49^	“We used three human PMSC lines (A, B and C) that were fully characterized following explant culture of three early gestation (14–21 weeks) placental donors as described in Lankford et al. as Donor 1, 2 and 3 [27].”
Jackson et al. (2021)^50^	“PMSCs used for this study were from one of our expanded product banks at passage 4. PMSCs in the cell banks were from consented donors that underwent rigorous screening and release testing”
Stokes et al. (2022)^51^	“The majority of our prior work evaluating PMSC-ECM for MMC repair in the fetal ovine model has been conducted using research-grade cell lines [12–14, 26]. In this study, we used clinical-grade PMSCs in preparation for a clinical trial. Placental tissue was collected from seventeen donors at the University of California, Davis.”
Theodorou et al. (2022)^52^	“The PMSCs were collected from a consented donor, with infectious testing of the donated placental tissue”
Farmer et al. (2026)^2^	“Human PMSC cell lines were generated from donated placentas collected from consented patients at the UC Davis Medical Center (CA, USA).”

These studies do not provide sufficient methodological detail to specify whether the PMSCs were sourced from donated placentas of miscarriages or of abortions. This lack of clarity is unfortunate, not only because it leaves the question of ethics unresolved but because it represents a failure on the part of the authors, reviewers, and editors to provide full methodological detail. In an attempt to clear up this lack of transparency, the study contact and contact backup listed on the clinical trial page^3^ were contacted by phone and email on March 27, 2026. As of the publication of this manuscript, a response has not been received, but due to the short timeframe, this lack of response has *not* been interpreted as dissimulative or concealing. If a response is obtained in the future, this manuscript will be updated accordingly.

In lieu of clarity from the investigators, a deeper dive is required to answer the question of PMSC sourcing. There is another study out of the same lab that describes the placental collection in the following terms: “Human placental tissues (n = 4) from the first trimester gestation (≤12 weeks of gestation) were collected from healthy consenting patients during elective abortions at the UC Davis Medical Center, with approval from the Institutional Review Board.”^53^This study was not among those cited in the timeline figure of Farmer et al., 2026^2^ but an argument could be made that there was likely much more work than could reasonably be included in a concise graphic such as this. However, it was funded by two NIH grants which were also used to fund several of the studies which *were *included in that figure^54,55^. While this does not prove that placentas used in the CuRe Trial were sourced from elective abortion, the totality of the circumstantial evidence is clearly suggestive of this conclusion. To summarize, the patents describe sourcing placental tissue from “terminated pregnancies,” the recent publication and publications leading up to it do not provide sufficient clarity to rule out elective abortion, and at least one publication from the same lab does explicitly describe sourcing placentas from elective abortion. Collectively, and particularly in the absence of transparency and clarity from the investigators, available evidence suggests that the pre-term placentas might have been sourced from elective abortions for this trial.

## 3. Where Does This Leave Us?

These revelations regarding tissue sourcing raise concerns regarding transparency and clinical research ethics. Due in part to the lack of clarity presented in the methods sections of these studies, it was not immediately evident that PMSCs were sourced from terminated pregnancies (and more specifically, potentially elective abortions). In fact, the supplemental video provided by ProLife Doc originally included with our review described the placental cells as being “harvested from the placentas of babies *after* those babies have been delivered,”^56^ a discrepancy which went unnoticed until this deeper dive into the methods. This video has since been replaced with an updated version without a voiceover. In light of this greater understanding of the CuRe Trial’s methods, three major questions arise that should be addressed:

1. The NIH considers placental tissue as HFT, and at least Hao et al., 2019 was NIH-funded^53^. How does the recent change in funding for HFT from elective abortion impact the CuRe Trial and similar work, if at all, assuming placentas from elective abortions are used?

2. Regardless of funding eligibility, what ethical alternatives exist or might be investigated to avoid use of placentas donated following elective abortion for these applications?

3. What, if any, scientific justification exists for using tissues sourced from elective abortion, rather than ethically uncompromised tissues from term or pre-term deliveries?

###  3.1. NIH Funding of HFT Research

In short, the NIH announcement ending funding for HFT research using tissue sourced from elective abortions does not impact the CuRe Trial, even if the placentas were from elective abortions. This is not because the NIH does not consider the placenta fetal tissue, as outlined above. Instead, it is because the NIH definition of research involving HFT from elective abortions specifically excludes “research on transplantation of HFT for therapeutic purposes (because of the statutory provision(s) addressing such research).”^36^ Such research is statutorily permitted^57^. A 2019 Congressional Research Service report claims that the NIH “does not currently fund any clinical research on human fetal tissue transplantation and has not done so for many years.”^58^NIH RePORTER shows the same^59^. However, as mentioned above, Hao et al., 2019 was funded by multiple NIH grants, both of which are correctly categorized as Transplantation, but neither of which are categorized as Human Fetal Tissue Transplantation^54,55^. Of note, one of the projects explicitly states the aim as being “to develop a novel fetal tissue engineering approach using stem cells and biomaterials” (which includes the placenta-derived PMSCs; i.e., human fetal tissue by the NIH definition^54^). Fetal tissue transplantation projects are categorized manually, and so it appears that (at least in this instance) the project has not been properly categorized, and that the broad claim that NIH does not and has not funded fetal tissue transplantation research is inaccurate.

 The answer to this question raises several others. How many miscategorized HFT Transplantation projects are the NIH funding currently or has it funded previously? Does the NIH have any intention to appeal to legislators to amend the statutory allowance of HFT Transplantation, continuing its trend of limiting funding for research involving HFT from elective abortion? Unfortunately, these questions are not as easily answered, and both remain unresolved.

### 3.2. Ethical Alternatives

If the PMSCs used in the CuRe Trial indeed are sourced from elective abortions, then it is not the ethically sound therapy that many had thought and hoped it was. So, what options exist or might be investigated that could serve as alternatives that do not involve intentional destruction of unborn lives?

 As mentioned above, it is theoretically possible that the “terminated pregnancies” after which these placentas were donated were in fact miscarriages. Regardless of the tissue source for this particular project, this remains an option for subsequent research and therapy. In fact, this trial provides a proof of concept of sorts for doing just that. With knowledge of the benefits that early gestation PMSCs can bring to spina bifida repair, researchers and clinicians can and should increase their efforts to solicit placental donation following miscarriage. Miscarriage is a tragic circumstance, and in an ideal world this would not be a potential source for these tissues because the miscarriages would not be happening. But as these miscarriages *do *occur, at the very least, this provides an opportunity to pull something good out of tragedy and save the lives of other babies. This is, admittedly, not as convenient or readily available a source of tissues as elective abortions. Another concern is that miscarriage is often associated with genetic abnormalities^60^, which could prevent the use of these placentas in certain cases. However, this source has the benefit of being deemed ethically acceptable to more people, so that in Dr. Bhattacharya’s words, “everyone is willing to take the kinds of progress” that are made.

Another option worth considering is further investigation of term placenta-derived PMSCs. These tissues are much more easily obtained ethically but would require more testing before efficacy could be demonstrated. When the CuRe Trial investigators compared the in vitro characteristics of the term and early gestation PMSCs, it was found that both cell types were positive for markers of mesenchymal stromal cells and negative for hematopoietic stromal cell and endothelial cell markers^45^. While it is true that the early gestation PMSCs exhibited certain benefits over term PMSCs (greater spinal cord tissue and neuron preservation post-repair, and improved motor function post-repair), this was an extremely small pilot study with only one lamb included in each repair group^45^. The early gestation PMSCs also expressed sex determining region Y-box 2 (SOX2), but this of course can, with optimization, be induced in vitro^61^. These term placenta-derived PMSCs should be investigated further, with larger sample sizes, in order to determine the feasibility of using this ethically sound tissue source.

If, however, term placenta-derived PMSCs are found to result in poorer outcomes after more rigorous testing, a placental biopsy could be taken from the patient early in pregnancy (as is done in chorionic villus sampling). Subsequently, autologous PMSCs could be sourced from this tissue, expanded, and used for the treatment later in gestation at the time of the spina bifida repair. Farmer et al. describe this in their paper but state that “[o]verall, previous studies suggested that allogeneic stem cells were a reasonable choice to test in human fetuses and potentially preferable to further exploring autologous sources, with the added benefit of sparing pregnant individuals the known procedural risks of chorionic villus sampling (which include a small chance of pregnancy loss).”^2^ Chorionic villus sampling indeed comes with risks, including possible miscarriage. These risks have been shown to be low, even negligible^62,63^, but of course must still be taken into consideration. However, returning to Dr. Bhattacharya’s point about public uptake of therapies involving unethical methods, accepting these risks would likely be more in line with the values and ethics of women who morally oppose elective abortion than utilization of PMSCs sourced from an abortion would be. These alternatives and their risks should be offered to patients for proper informed consent, and tissue sourcing information should be fully disclosed in clinical trial registries, trial consent forms, and scientific publications.

Additionally, it is worth remembering that the CuRe Trial utilizes an open hysterotomy repair technique (paired with application of the PMSCs). Separately, as discussed at length in our review^1^, fetoscopic approaches continue to advance. The PMSC-based approach has not been employed in humans long enough for a sample size large enough for any sort of head-to-head comparison of maternal and perinatal outcomes relative to fetoscopic approaches, but if placental tissue is unable to be sourced ethically, these minimally invasive, rapidly advancing, and increasingly safe and effective options do still exist.

Finally, the possibility of developing a similar (or novel) approach utilizing other, ethically sourced adult stem cells or iPSCs should not be discounted. Adult stem cells and iPSCs have both long been pursued as ethical alternatives to embryonic stem cells^20-24^ and adult stem cells have even been described as the “true gold standard in regenerative medicine.”^24^In the event that the NIH’s request for information^15^ results in a shift in funding from embryonic stem cells to adult stem cells and iPSCs, it is even more likely that such an alternative might be developed.

### 3.3. Abortion-Sourced Tissue vs. Ethically Sourced Tissue: Why the Former?

Perhaps the most important question, especially in light of the others discussed above, is what scientific justifications exist to continue utilizing tissues sourced from elective abortions rather than more ethical alternatives. Alternatives to tissues sourced from aborted embryos certainly exist^17-23^, and numerous avenues could be investigated further to improve and expand those alternatives. From an ethical standpoint, it is undeniable that a sizeable subset of the population takes issue with elective abortion, and with collection and use of tissues from those abortions. Indeed, a number of ethical arguments have been presented against the use of these tissues^17-21,64-67^. From a public health standpoint, it is worth reflecting on Dr. Bhattacharya’s quote above: Uptake and acceptance of scientific and clinical advances that seek to improve health will be hindered if patients do not find the means used to attain those advances ethical. From the scientific standpoint, is there reasoning that could not only justify use of these tissues but could also take precedence over these ethical and public health arguments? Several arguments have been offered over the years, too many to address point-by-point here. Dr. James Sherley has previously provided critical rebuttals to six such arguments, which the reader is encouraged to review^68^. One argument is worth mentioning briefly, because it is oft repeated. It has long been claimed that fetal tissue and embryonic stem cell research is critical for the understanding of and discovery of treatments for a number of debilitating disorders^69,70^. However, this argument has been made for decades, and the promises of miracle treatments and therapies have consistently failed to materialize^71^. Even as the CuRe Trial and other clinical trials utilizing fetal tissue or embryonic stem cells advance^2,3,72-75^, the collective therapeutic benefit derived from these sources is still drastically lower than the parallel benefit derived from ethical alternatives^17,22-24^. 

## 4. Conclusions

The CuRe Trial has been lauded as an exciting and innovative advancement in the field of fetal surgery, and indeed, it constitutes a very novel approach with promising preliminary feasibility and safety results. However, it is uncertain whether the placental tissues were sourced ethically, or rather sourced from elective abortions. While the NIH’s recent decision to halt research utilizing HFT from elective abortions will not impact this approach due to its status as transplantation research, it remains the case that the technique is seen as unethical by many. As Dr. Bhattacharya implied in his confirmation hearing, this results in a situation where regardless of the technological and scientific innovations that may come of it, their acceptance and uptake will be limited. Policies such as the NIH’s halting of HFT research funding seek to address this but leave projects such as the CuRe Trial unaddressed. In order to avoid forcing expectant mothers into ethical quandaries, policies of journals, clinical trial registries, and government agencies should ensure that the utmost transparency regarding tissue sourcing methodologies and available alternatives be presented. A mother receiving a diagnosis of spina bifida for her unborn child is already subject to distress and worry. It is our ethical imperative as researchers, clinicians, scientists, and innovators to not only require transparency, but also to encourage the development of ethical alternatives and ensure that her distress is not amplified by imposing a choice between the health of her baby and consistency with her ethical values.

## Highlights

*· Initial data from the groundbreaking CuRe Trial have recently been published, demonstrating feasibility and safety of the utilized approach.*

*· The National Institutes of Health (NIH) has recently announced changes to funding and requests for information regarding research using certain biomaterials, including fetal tissue and embryonic stem cells.*

*· The CuRe Trial, while not utilizing embryonic stem cells, introduces a new point of consideration in the scientific and ethical conversations surrounding the use of these biomaterials.*
